# Supplementary material for: Native dynamics and allosteric responses in PTP1B probed by high-resolution HDX-MS
Source: bioRxiv. 2023 Nov 13:2023.07.12.548582. Originally published 2023 Jul 12. Preprint. [Version 2] doi: 10.1101/2023.07.12.548582 (PMC10369962; doi:10.1101/2023.07.12.548582)
Supplement: Supplement 1 [file media-1.pdf]

Supplemental Information

Native dynamics and allosteric responses in PTP1B probed by high-resolution HDX-MS

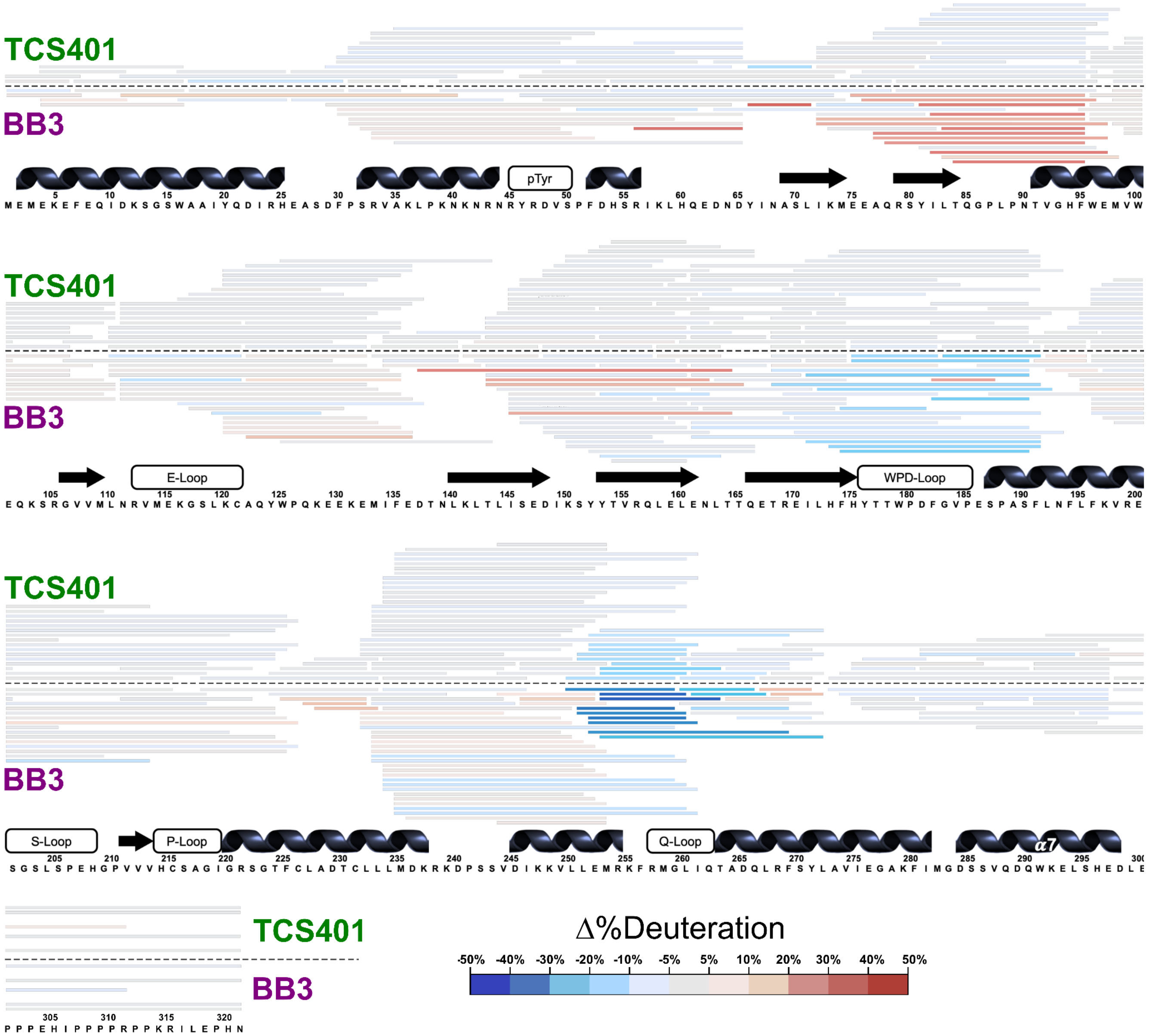

Figure S1: Difference HDX map for TCS401 minus apo PTP1B.
Peptide maps for the TCS401 and BB3 conditions, with peptides colored based on the difference in HDX rate between liganded minus apo conditions.

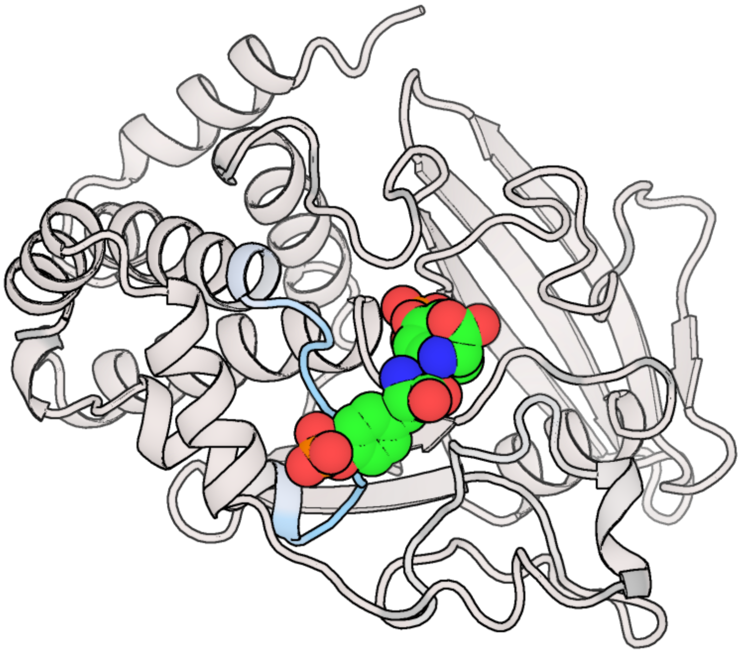

Figure S2: Structural view of TCS401 HDX differences in context of two pTyr sites.
Same as main **Fig. 5A** but with TCS401 omitted and the two pTyr residues from PDB ID 1PTY overlaid (green).

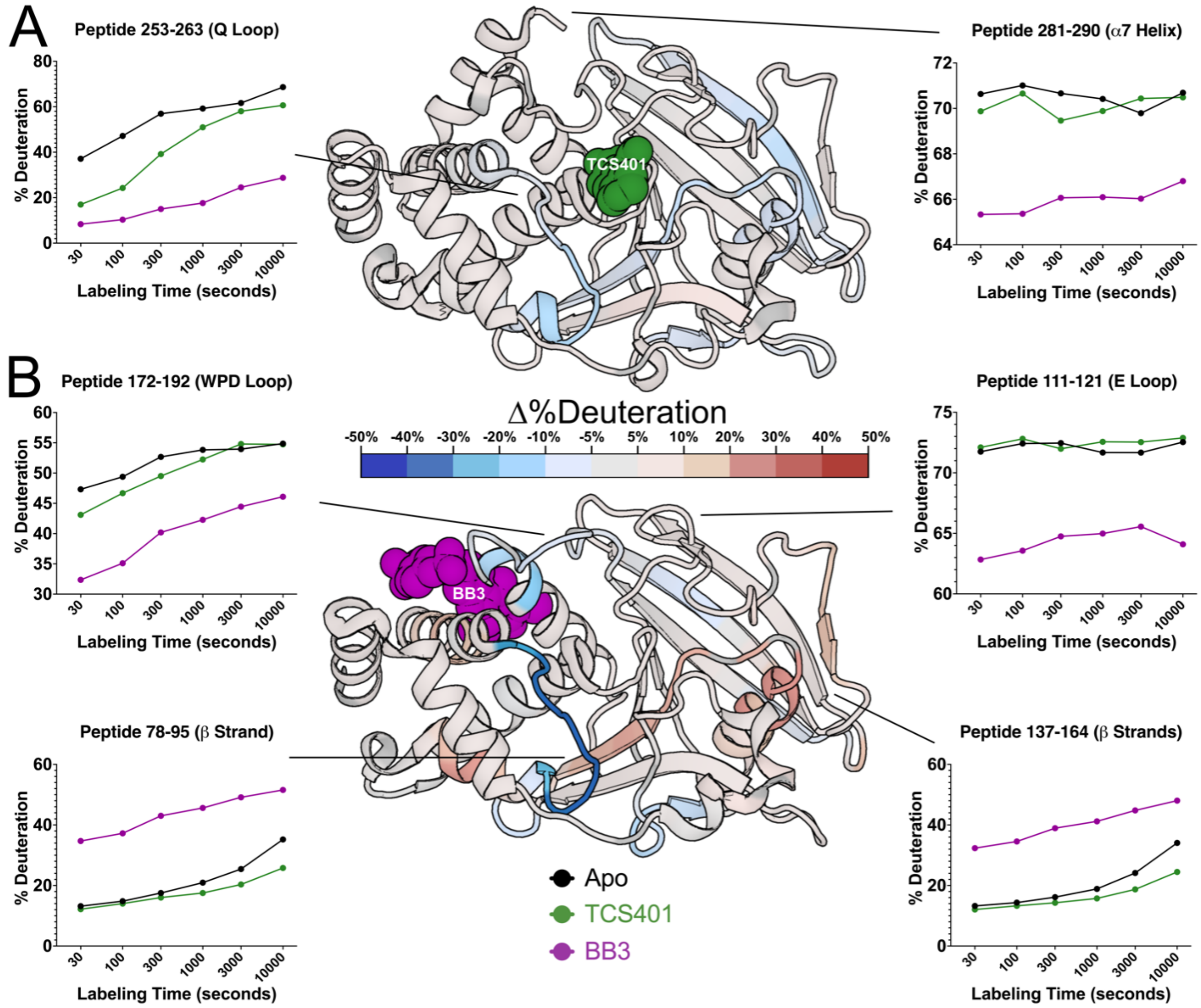

Figure S3: Effects of active-site inhibitor (TCS401) and allosteric-site inhibitor (BB3) after extended labeling time.
Same as **Fig. 5** but with coloring of residues at 10,000 seconds of labeling time.

Data S1: Fractional deuterium uptake for all three conditions.
Spreadsheet containing HDX and pseudo-ensemble data.
Tab 1: Peptide raw centroids for all three protein conditions across all time points.
Tab 2: Quantitative HDX data for apo PTP1B at 30 seconds of labeling time.
Tab 3: Cα RMSFs from pseudo-ensemble of PTP1B structures.
