# Supplementary material for: Native dynamics and allosteric responses in PTP1B probed by high-resolution HDX-MS
Source: bioRxiv. 2023 Jul 12:2023.07.12.548582. Preprint. [Version 1] doi: 10.1101/2023.07.12.548582 (PMC10369962; doi:10.1101/2023.07.12.548582)
Supplement: Supplementary file 1 [file media-1.pdf]

Supplemental Information

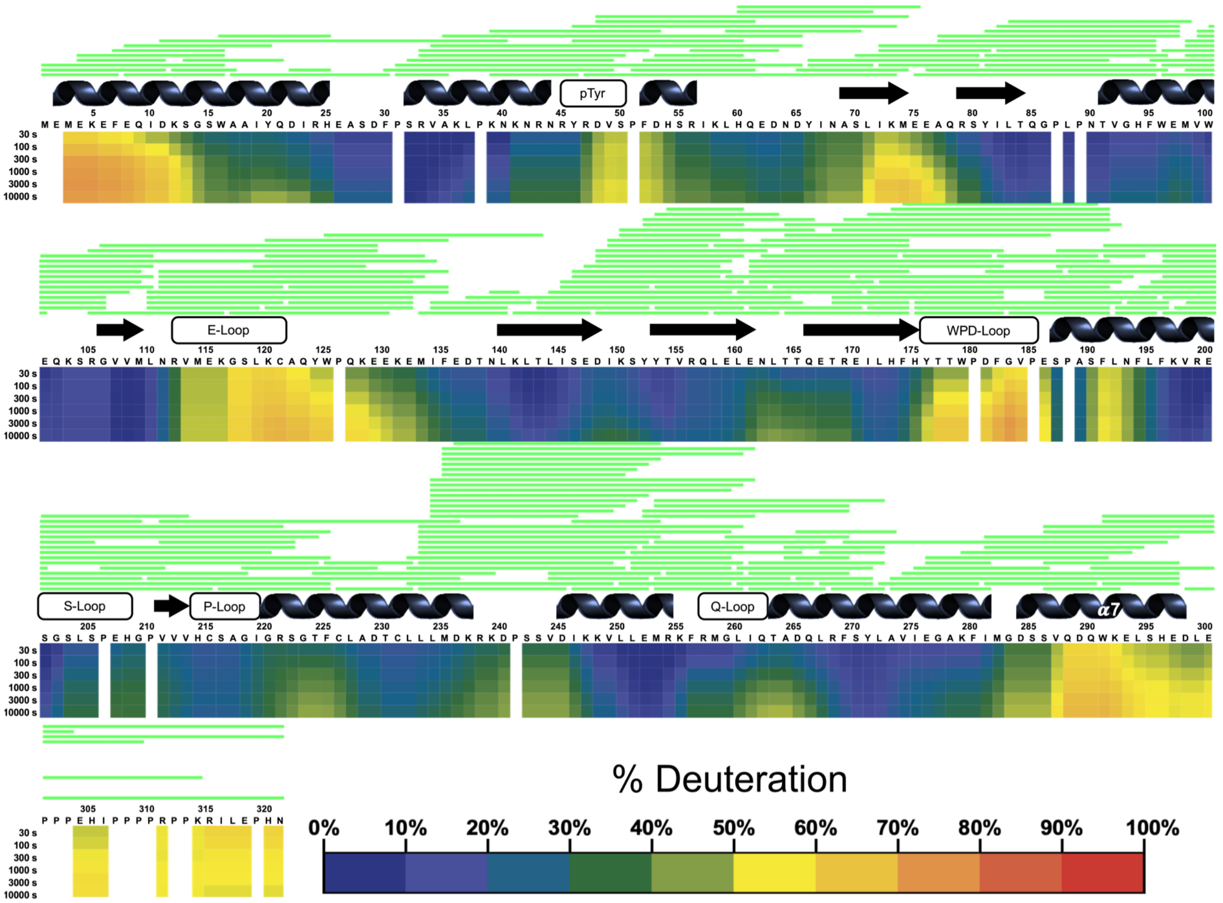

Figure S1: Local HDX map for PTP1B bound to TCS401.
Coloring and layout as in main **Fig. 2**.

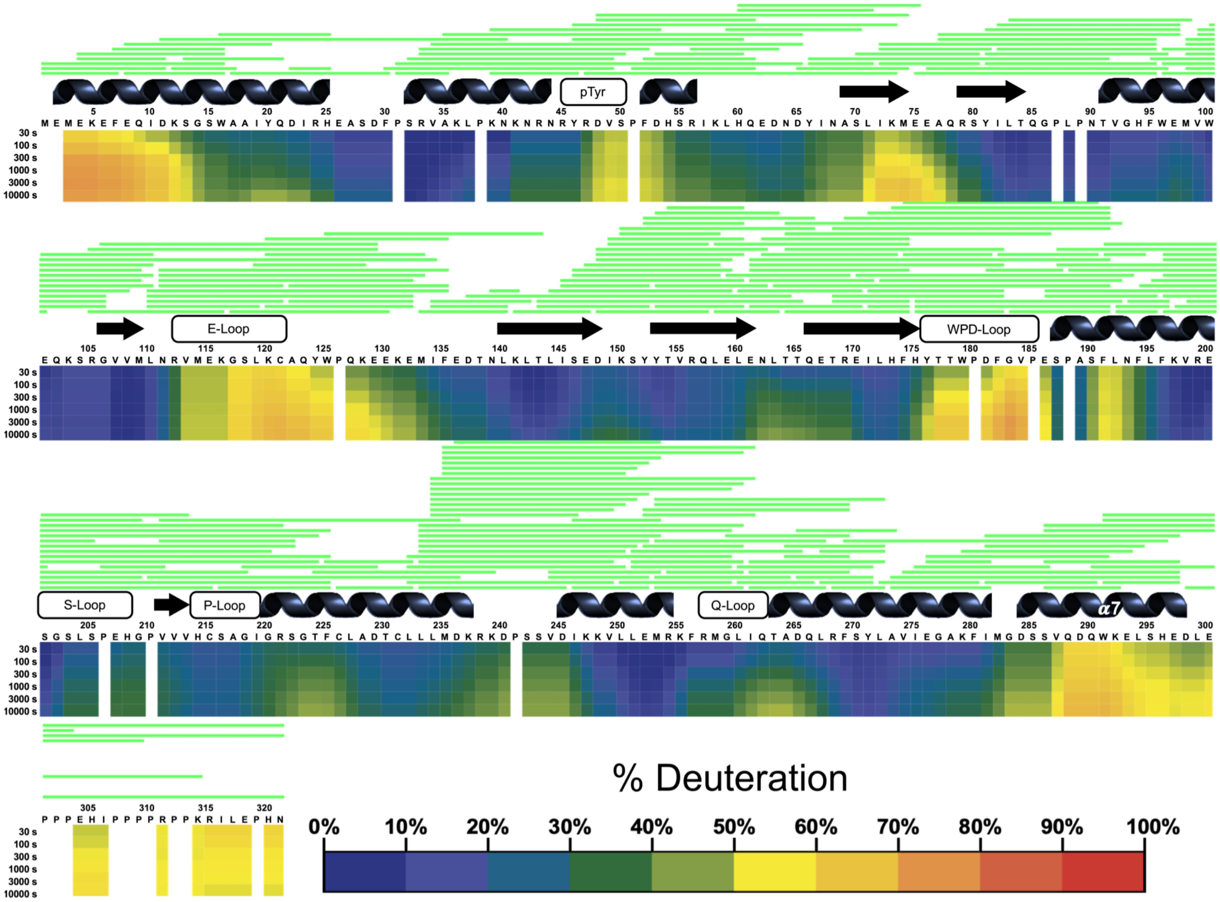

Figure S2: Local HDX map for PTP1B bound to BB3.
Coloring and layout as in main **Fig. 2**.

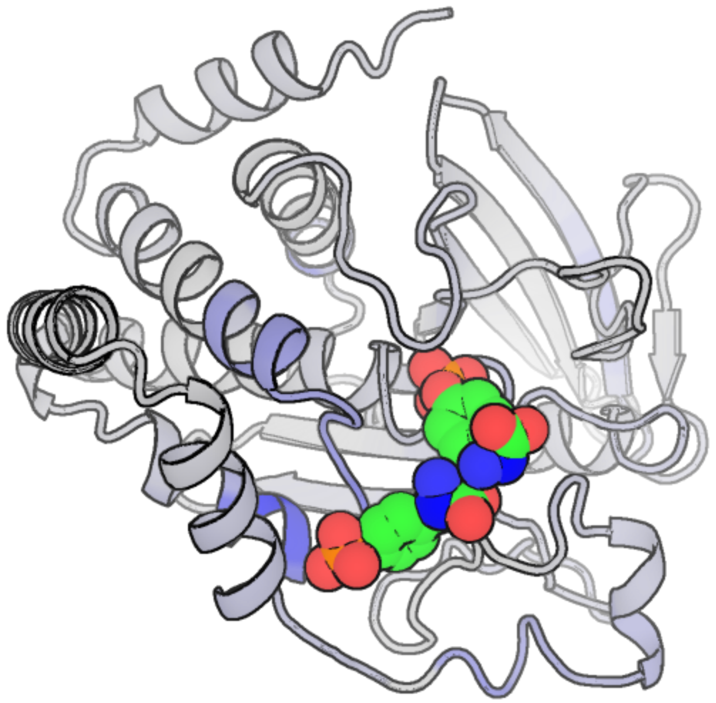

Figure S3: Structural view of TCS401 HDX differences in context of two pTyr sites.
Same as main **Fig. 5** but with the two pTyr residues from PDB ID 1PTY overlaid (green).

Data S1: Fractional deuterium uptake for all three conditions.
Fractional deuteration values at the residue level for apo, TCS401-bound, and BB3-bound PTP1B at all time points. “Spread” indicates the standard deviation of calculated fractional deuterium uptake values per amino acid or amino acid range. The spread is indicative of the variability among the calculated values based on the deconvolution of sometimes multiple peptides per residue, each charge species per peptide within an experiment, and results obtained across replicate experiments for the same time point.
